# Role of early life exposure and environment on neurodegeneration: implications on brain disorders

**DOI:** 10.1186/2047-9158-3-9

**Published:** 2014-04-29

**Authors:** Shweta Modgil, Debomoy K Lahiri, Vijay L Sharma, Akshay Anand

**Affiliations:** 1 Department of Neurology, Neuroscience Research Lab, Post Graduate Institute of Medical Education and Research, #3036, Research Block-B, 160012 Chandigarh, India; 2 Department of Zoology, Panjab University, Chandigarh, India; 3 Department of Psychiatry, Institute of Psychiatric Research, Indiana University Medical Center Indianapolis, Indianapolis, USA

**Keywords:** Aging, Metals, Epigenetics, LEARn, Methylation, Pesticides

## Abstract

Neurodegenerative diseases such as Alzheimer’s disease (AD), Parkinson’s disease (PD), Amyotrophic lateral sclerosis (ALS) and retinal degeneration have been studied extensively and varying molecular mechanisms have been proposed for onset of such diseases. Although genetic analysis of these diseases has also been described, yet the mechanisms governing the extent of vulnerability to such diseases remains unresolved. Recent studies have, therefore, focused on the role of environmental exposure in progression of such diseases especially in the context of prenatal and postnatal life, explaining how molecular mechanisms mediate epigenetic changes leading to degenerative diseases. This review summarizes both the animal and human studies describing various environmental stimuli to which an individual or an animal is exposed during *in-utero* and postnatal period and mechanisms that promote neurodegeneration. The SNPs mediating gene environment interaction are also described. Further, preventive and therapeutic strategies are suggested for effective intervention.

## Background

Early life plays an important role in health and development of an individual. Interactions between genes and environmental factors during early life are suggested to play role not only in human behavior but also in susceptibility to diseases. Surprisingly, in some individuals, onset of neurodegenerative disorders cannot be explained by family history. What triggers the sudden onset and rapid progression of these diseases still remains unexplained. Such sporadic diseases need to be studied in the context of early life environmental exposure. It is believed that environmental factors in childhood interact with the specific loci thereby modifying their expression and resulting in disease onset [1]. Epidemiological and animal based studies have also suggested a strong relationship between environmental factors and neurodegenerative disorders [2-8]. The effect of exposure to different environmental conditions during *in-utero* and developmental stages of life have been studied extensively and based on these studies various models have come into existence. A variety of agents including heavy metal exposure such as lead (Pb), manganese, mercury [9-11]; dietary habits [12,13]; pesticides [14-16]; stress [17] and other intrinsic factors such as inflammatory cytokines [18] affect early life and alter the regulation of gene expression. In this context, this review has been conceptualized to discuss the role of environmental cues that govern the onset of neurodegeneration. In addition, various single nucleotide polymorphisms (SNPs) associated with xenobiotic metabolizing enzymes (XMEs) have also been explained which may be useful for instituting preventive measures for adverse environmental stimuli.

### Environmental factors in neurodegeneration

It is widely believed that environmental constituents such as food, metals, pollutants, microorganisms and lifestyle play a direct or indirect role in brain health. For example, environment to which a fetus is exposed during the gestational period plays a significant role in future health of an individual. Postnatal period is also crucial for rendering an individual susceptible to environmental influences. Adverse prenatal and postnatal environmental conditions disrupt the homeostasis and increase the risk of neurodegenerative disorders. Various animal and human studies have been discussed in this context.

### In-utero conditions

Maternal environment affects the growing fetus as during *in-utero* stages, mother’s body is the only environment to which fetus is exposed. Growth of fetus is generally proportionate to the mother’s size and maternal constraint refers to the restriction provided to the growing fetus due to mother’s body size [19,20]. The maternal restriction affects growth by limiting the size of placental connection between mother and fetus thereby affecting the supply of nutrients for growth. The restraint is increased with age of mother, short stature and multiple pregnancies [21].

#### Human studies

Human fetuses are generally exposed to chronic placental insufficiency (CPI), hypoxia, heavy metals or hormonal disturbances in the mother’s womb. Studies have revealed that the chronic placental insufficiency (CPI) or umbilical cord occlusion to which fetus may be exposed to result in fatal hypoxenima [22] leading to synaptic dysfunction that triggers damage in neonates resulting in neurodegeneration [23]. Maternal hormonal disturbances also have adverse effect on fetus. Hormonal levels in fetus may be elevated if placental barrier between mother and fetus is compromised. For example, stress in mothers elevates glucocorticoid levels which travel through placenta adversely affecting fetus by programming the hypothalamus-pituitary-adrenal (HPA) axis due to change in number and affinity of glucocorticoid receptors in fetus [24]. Human studies showing the effect of gestational or *in-utero* exposure on neurodegeneration are limited. Most studies are either retrospective in nature, which imposes a recall bias in the study design, or if longitudinal studies are planned they are not of long duration.

#### Animal studies

As compared to human studies, animals provide an excellent model for longitudinal analysis of early life exposures due to comparatively small life cycle, easy maintenance and trackable follow up. Rat model of perinatal asphyxia has shown to affect retinal development by reduction in number of ganglion cells due to degenerative changes which lead to long term effects [25]. Similarly placental insufficiency was found to be associated to brain damage by impacting metabolic processes in rabbits [26]. Various mechanisms have been extensively reviewed by Johnston and coworkers [27] emphasizing that the developing brain is more vulnerable than the adult brain to the same insult. In an interesting study, pups of female exposed to lipopolysaccharide (LPS), a bacterial endotoxin, during pregnancy showed loss of dopaminergic neurons. This suggests that high LPS levels in mothers might interfere with the dopaminergic neurons in the fetus enhancing the susceptibility to PD [28]. Similarly, gestational exposure to metal toxins resulted in altered levels of various antioxidant enzymes in rats leading to oxidative stress [29]. Maternal hormones effect on newborns was reproduced in *in-vitro* studies on cerebral granular cells extracted from one week old pups of pregnant rats treated with dexamethasone and it was shown that oxidative stress due to glucocorticoids in cerebral regions is associated with neuronal apoptosis [30].

Together these studies not only highlight the importance of *in-utero* conditions in determining the health of fetus but also present an opportunity to increase the research investigations in this field of research.

### Dietary exposure

Dietary habits have significant effect on the physiology and metabolism of an organism. Growth and development of fetus is dependent on nourishment which is provided by the maternal system, thus, any food restriction during pregnancy has a direct or indirect role on fetus development. Deficiency or excess of any nutritive supplement to the mother results in long term consequences to the offspring.

#### Human studies

The possible effect of fetal nutrition on the risk of degenerative disease in later life has generated interest in 1990s resulting in extensive studies which elucidated the positive relation between diet and disease onset [31]. Positive relation between maternal diet and neurodegeneration has been supported in some human studies. Vitamin B-12 for example, is important for maintaining homeostasis in body and studies have shown that Vitamin B-12 deficient diet to mother during pregnant adversely affects the myelination in nervous system of offspring [32]. Postulating the role of maternal micronutrients, Roy and coworkers have demonstrated that imbalanced micronutrient supplementation in mother affects the level of antioxidant enzymes in the offspring increasing the risk of neurodegenerative diseases [33].

#### Animal studies

Similar to human studies, correlation between maternal diet and fetal neurodegeneration was reported in animal studies as well. Performance in Morris maze experiments is affected in pups born to mice fed on high fat diet during gestational and lactation period and the results were attributed to decreased cell proliferation [34]. Similarly, studies have shown that maternal folate depletion results in oxidative stress and epigenetic changes in the offspring [35] which ultimately lead to neurodegeneration. Further elevated levels of homocysteine in mother were shown to increase oxidative stress in pups brain leading to apoptosis, as marked by DNA fragmentation [36]. High dose of iron at neonatal stage has similarly been shown to result in neurodegeneration of midbrain at a later age. Pups with higher iron dose reduce dopaminergic neurons at age of 24 months as compared to that of 2 months old pups. This indicates that there are long term effects of neonatal iron exposure which are associated with degenerative changes [37]. Conversely, omega-3 fatty acid rich maternal diet is neuroprotective. This was shown by a study where omega 3 fatty acid supplementation to mother resulted in neonate protection from LPS induced brain injury [38]. Therefore, balanced diet during pregnancy has been suggested to protect offspring from neurodegenerative diseases.

### Metal exposure

Heavy metals consist of toxic pollutants pervading the environment. They are widely distributed in the environment and poison the living systems, as they accumulate. Mature tissue is protected from metal toxicity by the blood–brain barrier which prevents the movement of heavy metals from the systemic circulation to brain and by the formation of metal-protein complexes rendering metals unavailable to exert its toxic effects. In fetal brain this sequestering mechanism is impaired [39].

#### Human studies

Various metals such as aluminium, zinc, iron, copper and mercury have been linked with the neurodegenerative diseases. However, in some cases results are controversial and no direct association between these metals and neurological diseases have been demonstrated. For example, high level of aluminium in drinking water has been shown as a risk factor of Alzheimer’s disease in some studies while other studies fail to establish any such relation [40,41]. The reason for such contrary results includes inadequate aluminium analysis methods, improper selection of subjects and matching controls [42]. Transition metals like zinc and copper are other sources of brain toxicity and are believed to results in Aβ aggregation [43]. Like brain, retina is considered to be an immune privileged site due to presence of the blood-retinal barrier and has been found to be sensitive to metal toxicity. Metal exposure and its association with retinal degeneration has been examined in various studies [44-46]. Low and moderate level of gestational lead exposure (GLE) i.e. first trimester results in increased amplitude of a and b waves in 7–10 year old children [47]. Similarly high level of mercury and Pb in umbilical cord blood due to prenatal exposure impaired the visual processing as shown by visual evoked potential measurement in exposed children after 11 years [48].

#### Animal studies

Toxic effects of heavy metal exposure are also evidenced from animal studies. Long-term potentiation (LTP) which is responsible for enhancing the signal transmission between the neurons is considered as the major mechanism underlying information storage and memory formation, resulting in increased synaptic strength [49]. Enhancement in signal strength is dependent on two factors, one is the presynaptic increase in neurotransmitter release and other is enhanced function of glutamate receptor at the postsynaptic end. NMDA receptor function has been found crucial for the LTP induction in hippocampus [50,51]. Neonatal exposure to aluminium chloride has been shown to reduce the LTP amplitude in rats by affecting both presynaptic and postsynaptic signal transmission [52]. Heavy metal exposure such as zinc, copper and Pb have a negative effect on LTP during developmental stage as it reduces the potentiation magnitude and increases its decay time as well as the threshold level for induction in hippocampus [53,54].

Combined prenatal effects of arsenic, cadmium and Pb in rats exposed to metal mixture have been shown to disrupt blood–brain barrier and cause memory deficit [55]. Although various studies have focused on the role of different metals in pathogenesis of neurological disease, the role of Pb is most widely investigated. The early life exposure of Pb and its effect on adults has thus been a major area of investigation for past few years. Rats exposed to low Pb level during *in-utero* and lactation period have shown impaired learning and memory, hyperactivity and anxiety in adults [56]. *In vivo* studies of Pb exposure on various animal models, such as rats and monkeys, have revealed the role of developmental exposure of sub-toxic doses of Pb on neurodegeneration. It is evident from studies that the Pb exposure in developmental stages results in the increased level of beta amyloid in brain causing Alzheimer in later age [57,58].

### Pesticides

Pesticides are other major pollutants or toxins to which living organisms are exposed. Health issues related to pesticides prevalence in environment are of major concern. These pesticides include insecticides, herbicides and fungicides. Insecticides such as organophosphates, organochlorines and carbamates are used more frequently and enter the living system through respiratory tract, gastrointestinal tract or through dermal contact [59,60]. Ocular exposure, although not a common route of exposure, may occur through accidental splashing of pesticides into eyes or through contact of hands with eye and further from ocular tissue to blood circulation [61]. β radiation based radioactive studies have revealed movement of carbamate from the cornea to the retina via aqueous humor supporting the exposure of pesticide through ocular route [62].

#### Human studies

Exposure to pesticides is more prevalent in individuals working in agricultural sectors such as farmers, peasants, farm workers. They are at increased risk of direct exposure while others may be exposed due to food contamination [63]. Contaminants get accumulated in the body and change the gene expression profile in exposed tissues. Pesticides are thus believed to be one such contaminant that can alter the regulatory framework and lead to disease onset and progression through epigenetic changes [64]. Pesticide exposure has been shown to result in neuronal loss, cognitive impairment and motor dysfunction. These alterations in neurological behavior may be associated with neurodegenerative diseases.

#### Animal studies

Pesticides exposure studies in animals supported the adverse effect of early life exposure on later life. It was evidenced from study in which exposure to dieldrin during gestation and lactation has been reported to affect the dopaminergic responses in offsprings. Exposed mice showed elevated level of dopamine transporter and vesicular monoamine transporter 2 (VMAT) proteins. These alterations were persistent through later stages in life leading to dysfunction of dopamine making dopamine neurons more susceptible to damage in adulthood [65]. Another pesticide, paraquat in combination with maneb, has also been shown to be more destructive in animal studies and leads to PD by dysfunction of nigrostriatal dopaminergic system as well as motor response abnormalities [66]. Likewise, permethrin, when administered to rats at age of 6–21 day results in glutamate, NO and calcium imbalance in brain hippocampus [67]. Despite accumulating evidence of the effect of pesticides in pathogenesis of neurodegeneration, very only fewer studies have integrated this aspect of investigation in understanding of brain disorders.

### Lifestyle, smoking and drug abuse

Lifestyle plays a central role in health and well being of organisms. With increased sedentary lifestyle and lack of physical activity the incidence of diseases is also increasing. Healthy lifestyle prevents disease occurrence whereas bad habits increase the susceptibility to disease. Exercise, in particular aerobic exercise, has a positive impact on brain functioning.

#### Human studies

Importance of healthy lifestyle in human life has been demonstrated. Childhood aerobics increases the resilience of the brain in later life [68]. Similarly, the association of caffeine, smoking and alcohol consumption has been well reported in neurodegenerative diseases [69-71]. Our SNP studies with patients of age related macular degeneration (AMD) showed higher frequency of TT genotype of CCL2 gene. Interestingly, the frequency of TT genotype was found to be higher in smoker AMD patients when compared to nonsmoker AMD patients [72] highlighting the role of smoking in exacerbating the pathogenesis of disease. Early life exposure to smoking with degenerative disease has not been investigated adequately and could be the subject of future research projects.

#### Animal studies

**Figure 1:**
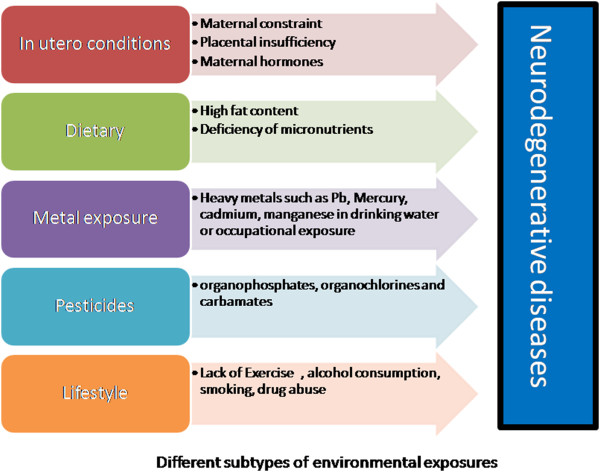
Different subtypes of environmental exposures.

Studies carried out on animals further strengthen the correlation between lifestyle and neurodegeneration. In a study, the pups born to mothers underwent low intensity treadmill exercise during pregnancy were shown to have more hippocampal cell survival [73]. Similarly, pups performing treadmill exercise at postnatal day 21–60 showed enhanced spatial memory as compared to controls [74]. Drugs such as methamphetamine (MA), which is widely abused due to comparatively low prices in comparison to cocaine or heroin [75] have been studied for its role on retinal damage in rats born with prenatal and postnatal methamphetamine exposure. Female rats exposed to MA at gestational stage have shown altered optic nerve patterns in newborns with optic nerve diameter smaller than the controls. Furthermore, it has also been reported that optic nerves of MA exposed rats have reduced production of myelin basic protein and increased number of deformed axons, mean optic fiber area, less lamellar separation [76-78] (Figure 1; Table 1).

**Table 1:** Spectrum of environmental stimuli and their effects on neurodegeneration

**S. no.**	**Exposure**	**Subject/animals**	**Period of exposure**	**Effect**	**Reference**
1.	Ethanol	Mice	Postnatal day 3-20	Decreased number of neurons in Retinal ganglion cell layer and dorsalateral geniculate	[79]
2.	Microwave irradiation	Mice	Prenatal + 4 months postnatal	Complete degeneration of RPE, nuclear pyknosis in photoreceptors, thinness of all layers	[80]
3.	Fried potato chips	Rats	Gestational day 6-postpartum day14	Vacuolization and apoptosis in GCL, swollen choriocapillaries, alteration in cellular organelles	[81]
4.	Lead (Pb)	Mice	Lactation period	Altered mitochondrial morphology, mitochondrial phosphorylation dysfunction	[82]
5.	Rotenone	Rats	Postnatal	Thinness of GCL, disruption of mitochondrial complex I, photoreceptor loss	[83]
6.	Cycus plant		Postnatal	ALS and PD	[84]
7.	Pesticide contaminated drinking water	Human	Postnatal	Inhibitory effect on antioxidant enzyme systems, mitochondrial and proteosome function (PD)	[85]
8.	1-methyl-4-phenyl-1,2,3,6-tetrahydropyridine	Mice	Gestational day 8-12 and postnatal	Apoptosis of nigrostriatal dopamine neurons enhancing toPD risk	[86]
9.	Methamphetamine	Mice	Postnatal day 11-21	Altered level of muscarinic acetylcholine receptors in the hippocampus	[87]
10.	Cypermethrin	Rats	Postnatal day 5-19	Dopamine, 3,4-dihydroxyphenylacetic acid (DOPAC) and homovanillic acid (HVA) level in brain altered	[88]
11.	Aluminium	Mice	Pregnancy day 1-15	Neurotoxicity by affecting dopaminergic system	[89]
12.	Tobacco inhalation	Mice	Gestational day 6-17	Altered gene expression profile affecting morphology and function of hippocampus	[90]

### Mechanism, hypothesis and models

#### Epigenetics

Recent studies have focused on epigenetic mechanisms that modify the onset, latency period and progression of neurodegenerative diseases [91]. Epigenetics is an emerging field that focuses on the mechanisms that alter the function of genes. It generally takes into account the gene and environment interaction such that these changes are inherited. The epigenetic changes do not involve alteration in nucleotide sequences in the DNA but influence its functioning by controlling its expression by gene reprogramming [92]. The epigenome is therefore considered different from genome in being dynamic. It is altered by environmental signals, not only during the period of exposure but even later in life. It has been shown that fetal epigenetic patterns can be altered at later stages by environment exposures [93,94]. A traditional insight into the field is exemplified by the example of identical twins having same genotype but possessing different epigenetic patterns in adulthood due to different environmental exposures leading to different epigenome and disease susceptibility [95-97]. Epigenomic variation leads to phenotypic diversity as well as susceptibility of individuals to disease. These changes are generally brought about by environmental influences. DNA methylation and histone acetylation have been recognized as epigenetic processes which regulate the functioning of gene. Histone acetylation controls the heterochromatic and euchromatic state of DNA wrapped around histones, and remaining in dormant state. Histone acetylation unwinds the DNA from histone and renders it available for transcription. Along with histone acetylation, DNA methylation plays an important role in regulating accessibility of DNA for transcription. Histone acetylase transferases (HAT) and Histone deacetylase (HDAC) controls histone modification in cell [98]. Animal studies have been used to describe the epigenetic pathways of disease etiology. It has been demonstrated that the early life exposure to various environment stimuli leads to methylation pattern changes in promoter region, resulting in altered gene expression in later stages. Methylation patterns have been found to be altered in mice offspring by methyl donors or low proteins in mother’s diet [99]. Some sites in the genome are more susceptible to the epigenetic changes. It is, therefore, pertinent to note that C_p_G islands are targeted more often for methylation [100]. Thus, switching on and off of expression is under the control of epigenetic patterns of histone acetylation and DNA methylation changes [98] which are influenced by early life exposure.

**Figure 2:**
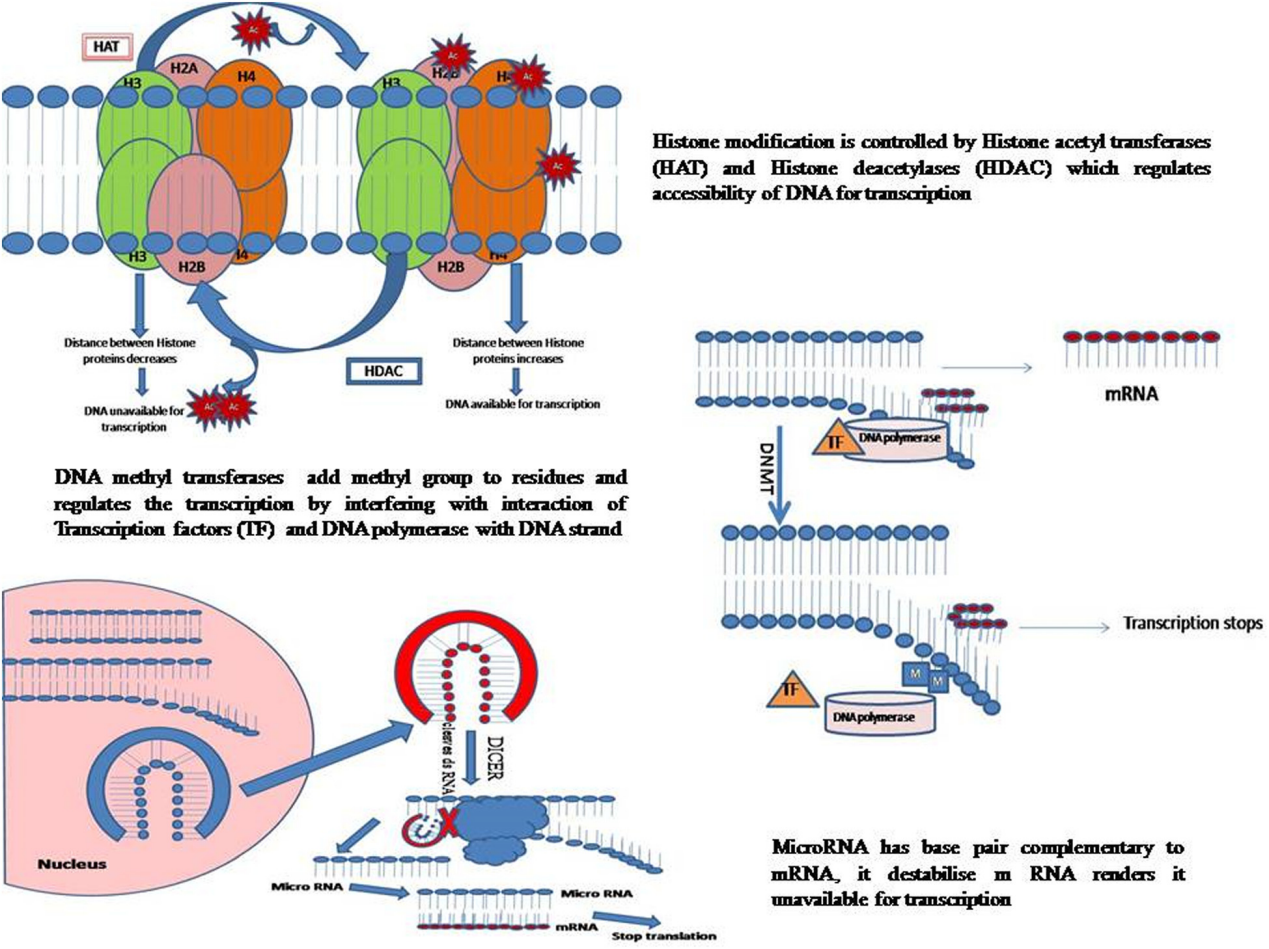
Epigenetic processes that regulate the gene expression at transcriptional and post-transcriptional level.

The non-coding RNA referred to as microRNA is believed to act at post transcriptional stage thereby exerting epigenetic regulation of such changes. MicroRNAs control the gene expression by interfering with the mRNA thereby destabilizing it and rendering it unavailable for translation. This unique property enables it to regulate many different mRNAs [101] (Figure 2).

#### Barker hypothesis or fetal basis of adult diseases (FeBAD)

Barker and coworkers have proposed the FeBAD model after their studies on adult cardiovascular diseases and their fetal origin [31]. According to Barker’s hypothesis adult diseases are more or less consequences of fetal adverse conditions. Although Barker’s work was mainly confined to cardiovascular diseases, the hypothesis fits well to other diseases too. The fetus gets adapted to new environment depending on environmental stimuli in uterus by means of physiological and hormonal alterations and prepares itself to the upcoming conditions in postnatal life, a phenomenon called fetal programming. It takes cues from the maternal health status and show adaptive responses to survive in the maternal environment. Adaptive responses may be either in the form of metabolic changes, hormonal release or sensitivity of the target organs to hormones, which in turn affects the development of target organs, leading to physiologic and metabolic disturbances. Thus, the reduced growth or body size can be considered as a fetal adaptive response towards small uterus size of mother with no immediate consequences in the newborn but which may lead to physiologic changes that can cause diseases in later life [19].

#### Developmental origin of health and disease (DOHaD)

The DOHaD model was a modified version of FeBAD which postulated that postnatal period of development also plays an equal role as fetal life in health. According to DOHaD, the adaptive responses during developmental stages, which include not only embryonic development but also the period of development during infancy, are responsible for late life risk of diseases [20]. Environmental conditions prevailing during the infancy phase exert their influence on the genotype and alter the organism’s ability to cope with its environment in later life. As compared to intra-uterine environment, which remains relatively constant throughout gestation, postnatal environment changes drastically. The DOHaD phenomenon explains how changing environmental factors affects the patterns of diseases.

#### Predictive adaptive response (PAR)

Gluckman and Hanson have suggested that when fetus is exposed to adverse conditions or stress it makes immediate changes which are often reversible, but if the stress conditions are prolonged, fetus undergoes irreversible changes which then persist throughout life and influence the adulthood. They coined term PAR for the phenomenon. The fetus predicts the extra-uterine environment from intrauterine conditions and makes changes for its better survival. These irreversible changes may or may not be useful to the fetus in the long run. If extra-uterine environment will be different from intrauterine, it will suffer from the physiological manifestations as changes in response to predictive environment will not match the actual environment [102]. If adaptations match the environment, then it leads to the better survival. For example, meadow vole pup born in autumn has thicker coat due to adaptive response to the signal emanating from maternal melatonin levels *in-utero* and thus has better survival [103].

### LEARn model

**Figure 3:**
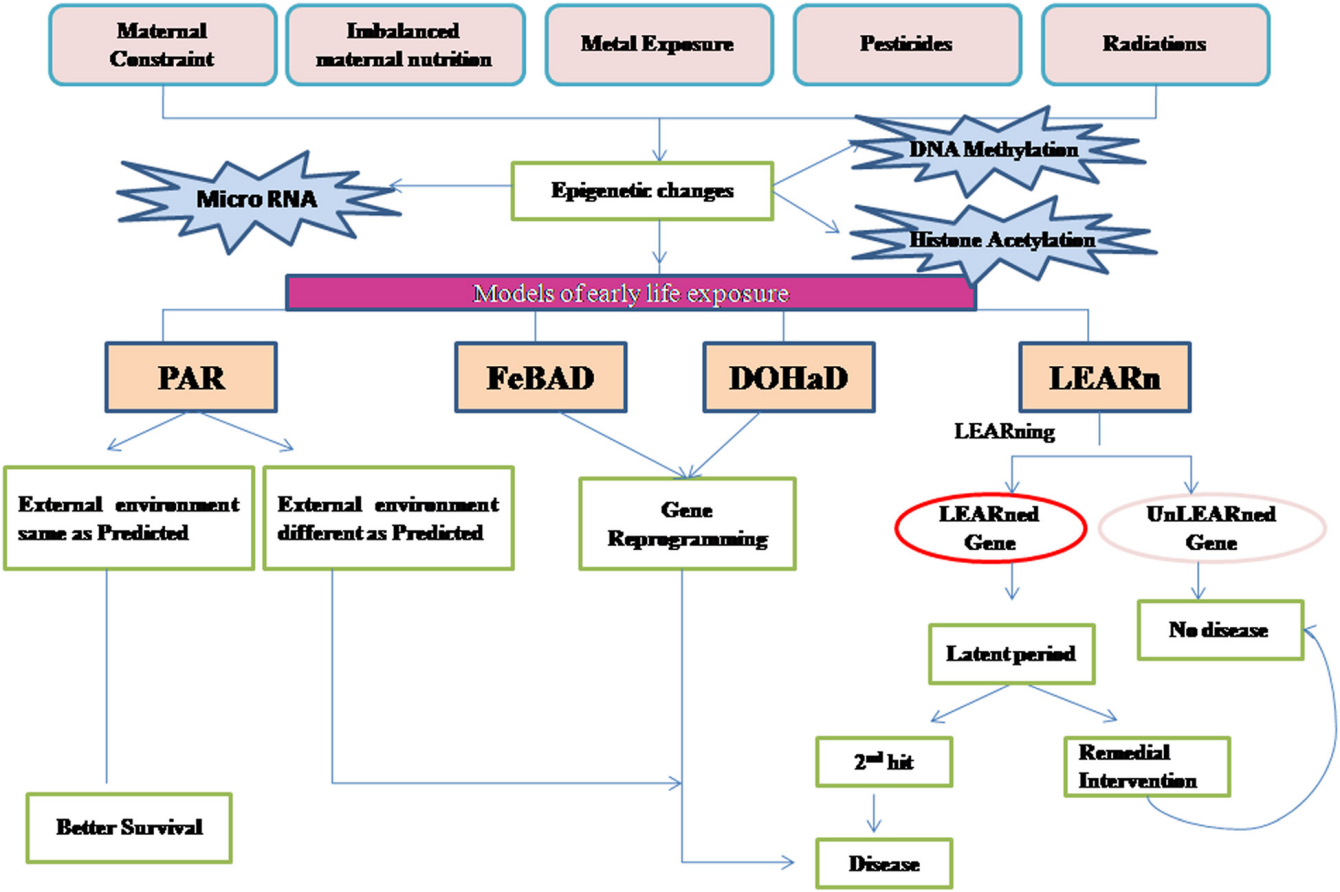
**Effect of environmental factors on late-life disorders.** The schematic summarizes the proposed mechanism and different models of early life exposures and how they operate.

LEARn (Latent early life associated regulation) model suggests the role of environmental factors in disease etiology. Lahiri *et al.*[94] have described the association of early environment with disease onset especially with respect to Alzheimer’s disease. Due to lack of knowledge pertaining to disease cause and progression, the sporadic onset of several diseases have been believed to be associated with many environmental agents such as nutrition [104], head trauma [105], metal exposure [106] and lifestyle [107]. LEARn model describes these environmental exposures as ‘hits’. The authors contrasted LEARn against different acute and chronic models of disease progression [94]. LEARn is distinct from these models in that it is neither acute nor chronic but acts through induced latent epigenetic changes. They further suggested that all neurodegenerative disorders come under the category of a ‘n’ hit latent model, according to which early life exposure leads to epigenetic perturbations in the genes but do not result in any disease symptom. A second trigger is required for the disease to develop and this time between first hit and disease onset is termed as latency period. Genes are divided into two categories the one which respond late in relation to early life responses (LEARned) and others which don’t (unLEARNed). The process of responding to the early life environmental triggers after the long latency period is termed as LEARning [94] (Figure 3).

### Prevention and reversibility

Reversal of induced changes may be possible if associated epigenetic (methylation, acetylation) and physiologic (gene expression) changes can be switched back to normal. Cognitive impairment because of imbalanced maternal diet has been tested by leptin treatment as leptin receptors are present in brain regions and known to regulate neuronal excitability and long term potentiation [108]. Peroxisome proliferator activator receptor α (PGC1α) regulates the expression of genes involved in bioenergetics. (PGC1α) expression in offspring of under-fed female rats returned to normal by exogenous supply of leptin [109]. Similarly folate deficiency related neurodegeneration is ameliorated by dietary S-adenosylmethionine (SAM) supplementation. Folate deficiency has been shown to result in neurodegeneration in mice due to reduced level of SAM which is attenuated by apple juice concentrate supplementation, containing high levels of SAM [110-112]. Likewise, polysaturated fatty acids exerts neuroprotective effect against neurodegeneration in PD and AD models by ameliorating the adverse effects of neuronal toxicity [113,114] and creatine rich diet has also been shown to sustain the harmful effects of birth hypoxia [115]. These studies highlight the possibility of restoring altered epigenetic changes and provide scope for instituting therapeutic approaches for ameliorating degenerative diseases. Remedial intervention during latency period can prevent the disease onset by reversing the abnormal conditions back to normal for e.g. complete degeneration of inner retina by early life exposure to monosodium glutamate (MSG) [116] has been found to be reversed by enrichment of postnatal living conditions in rats. Provision of appropriate housing conditions such as larger cage size readily reversed the effect of MSG on retinal thickness [117]. Exercise is another preventive measure that has been shown to modulate the expression of genes regulating the methylation and acetylation of DNA and protein. Studies have shown decreased expression of DNA methyltransferases [118] and increased expression of HAT [119] in the hippocampus of rats which exert their epigenetic influence by increasing the expression of neurotrophic factors in brain. Further evidence was provided by Scopel *et al.*[120] by showing that exercise regime of 20 minutes for 2 weeks for wistar rat attenuates the damage in hippocampal slices submitted to ischemia *in-vitro* opening the field for further investigation.

### Therapeutic interventions

While prevention is always better than cure, sometimes it is not feasible to prevent an environmental exposure due to occupational demand, as in pesticide exposure to farmers and metal exposure to workers in metallurgy industry is imminent. Similarly, if the sole source of water supply is contaminated, exposure to pollutants cannot be avoided. In such cases identification of targets for disease reversal are useful tools for pioneering therapies. The environmental agents modulate the normal functioning and physiology of central nervous system (CNS) by mechanisms that involve altered gene expression through modulation of signal pathways. These mechanisms, if explored, can provide a window of opportunity for therapeutic intervention during latency stage, thereby delaying or preventing the onset of disease. Recent studies have tried to elucidate the underlying mechanisms by which the environmental agents exert their toxic effects on CNS. Pb is reported to accumulate amyloid-β in brain tissue by decreasing the activity of insulin degrading enzyme (IDE) and neprilysin (NEP), both known for amyloid beta degradation [121,122]. Exogenous administration of IDE and NEP may thus provide a good approach to prevent the lead induced toxicity. Another key factor involved in neurodegeneration is oxidative stress as is evident from studies related to AD and PD [123,124]. Environmental toxins such as heavy metals act as an electron acceptor or donor and result in formation of reactive oxygen species, leading to oxidative stress [125]. Therefore, antioxidants can be used for metal intoxification due to their property of ameliorating the oxidative stress. Certain antioxidants such as α-lipoic acid and vitamin E have already been reported to prevent neurotoxicity induced by copper [126]. Herbal extracts of Lutein*, Allium cepa,* and other natural antioxidants can similarly diminish the adverse effects of oxidative stress and prevent rapid disease progression [127,128]. By reducing the cause that results in neurodegeneration, remedial steps to reverse the effect can be evaluated. Metal exposure and drug abuse, for example, disrupts the signaling pathways, as manganese toxicity in striatum has been found to alter the AKT1/2 and ERK signal pathway [129] resulting in impaired VMAT and dopamine active transporter (DAT) regulation [130]. In such case the neuroprotective substance should be able to maintain the normal signaling pathway so that the expression of VMAT and DAT protein is not compromised. Trolox, has been found to reverse the adverse effect of manganese on ERK 1/2 pathway [130] while a Chinese prescription, Zhen Wu Tang (ZWT) ameliorates the neurodegenerative process by maintaining levels of VMAT-DAT mRNA [131]. Thus, both of them provide a therapeutic approach against metal toxicity. Similarly rotenone induced neurotoxicity was ameliorated by oxytocin by reducing the expression of various caspases which were responsible for apoptosis [132]. Likewise, targeting PGC 1α can be useful in PD patients as elevating PGC1α levels in *in-vitro* studies prevented dopaminergic neuron loss [133].

Metal induced neurotransmitters-receptor sensitivity and cause neurodegeneration. LTP has also been suggested to result from the malfunctioning of NMDA receptor [134,135]. NMDA receptor is a hetero-dimeric structure and the functionality of receptor depends on the proper assembly of subunits. Expression of NR2A subunit of receptor has been reported to be reduced due to Pb exposure resulting in altered LTP suggesting that NR1/NR2A receptor complex is required for the calcium mediated signaling to maintain the cognitive ability [136]. Taurine supplementation on the other hand was found to be protective against NMDA receptor malfunctioning by reducing calcium overload [137]. Therefore, for diseases related to NMDA receptor malfunctioning and calcium influx, taurine can be considered as neuroprotective.

**Figure 4:**
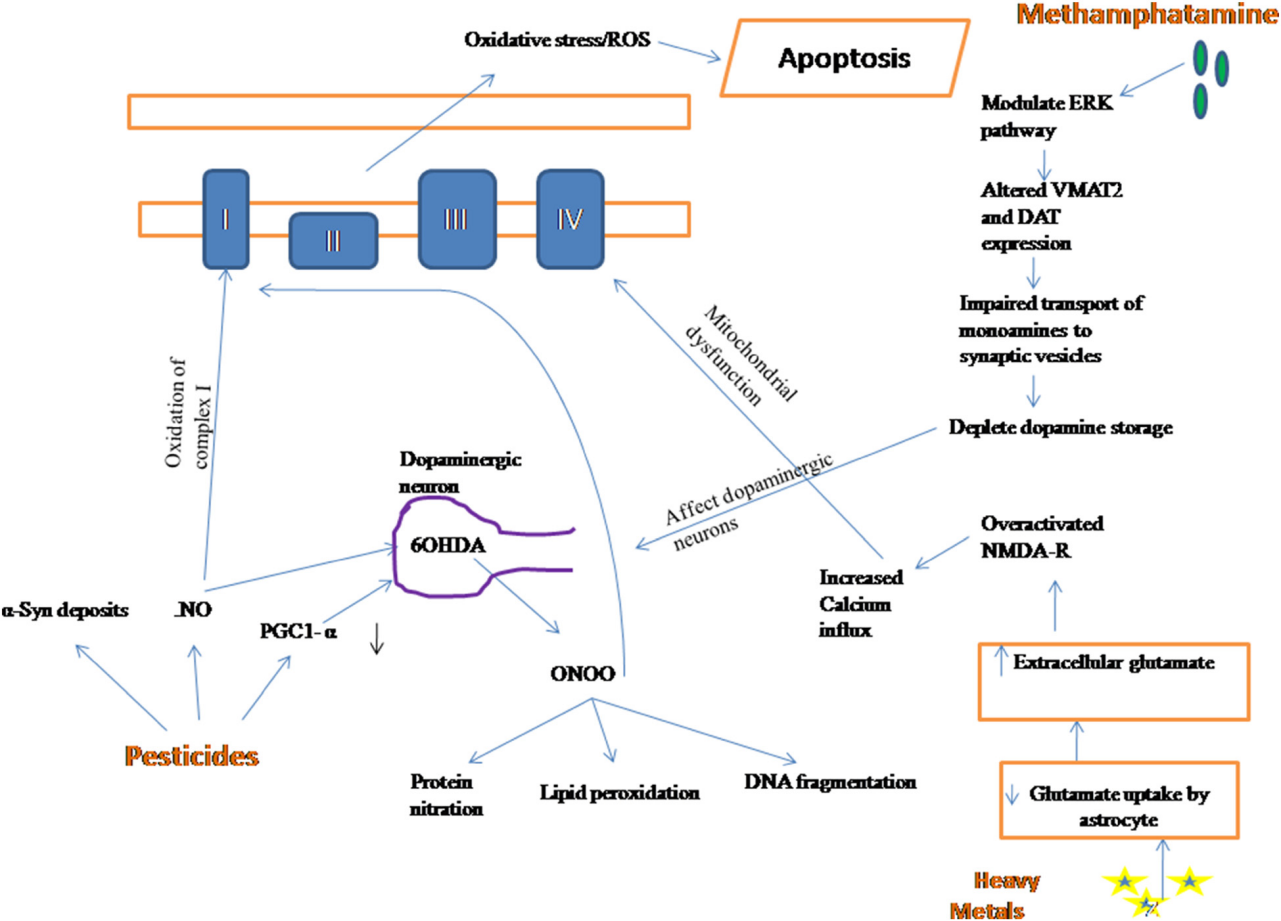
Schematic diagram representing the action of different environmental stimuli (metals, drug and pesticides) on dopaminergic neurons, glutaminergic neurons and mitochondria leading to neuronal apoptosis.

Ubiquitin Proteosome Complex (UPC) maintains protein homeostasis in the body by degrading the misfolded, malfunctioned and accumulated proteins and inhibition of UPC results in aggregation and deposition of these malformed proteins in CNS leading to neurotoxicity [138]. As also described in epigenetics section that histone modification plays a major role in regulation of gene expression, HDAC inhibitors such as valproic acid, trichostatin and phenylbutyrate have been found to be neuroprotective. They exert neuroprotection by regulating the expression of neurotrophic factors such as glial derived neurotrophic factor (GDNF), brain derived neurotrophic factor (BDNF) and reducing inflammation and neuronal death [139,140]. Thus, therapeutic intervention by targeting these known processes can also prevent the progression of disease from environmental hazards. These neuroprotective agents thus help in disrupting the cascade of reactions that ultimately lead to cell loss by apoptosis (Figure 4).

### Genetic susceptibility to environmental stimuli

Individuals exposed to same environment respond differently and this difference is attributed to differences in genetic make-up. SNP studies focus on the polymorphisms in genes which influence the susceptibility of individual to the environmental stimuli. Studies have been carried out to show that risk to the environmental toxins such as heavy metals and pesticides have positive correlation with gene polymorphisms. Polymorphism in XME genotype influences the metabolizing efficacy of enzyme. SNP variation effect the normal functioning of enzyme by altering the enzyme kinetics. One allele of glutathione synthetase *(GSS)* was found to be more interactive with metals over the other enhancing the risk of toxification [141]. Similarly, glutathione transferases *(GSTs)* are another group of enzymes involved in detoxification processes by ubiquitinization of pesticides and other toxicants. *GST* genotype and heavy metal metabolism have been studied and it was found that one form of gene readily metabolizes metals into non-toxic form and thus reduces the risk of toxicity [142,143]. Children of mothers with *GSTM1* and *GSTT1* allele, prenatally exposed to pesticides are at greater risk of fetal growth restriction [144]. Further studies on this gene revealed the positive associated of gene polymorphism with AD, PD and AMD [145]. Similar genotype study on human paraoxonase 1 *(PON1)* enzyme revealed that one form of gene is associated with increased susceptibility to pesticide related damage. Children of mothers with susceptible genotype have been found to be more prone to toxicity due to prenatal exposure of organophosphates [146]. N-acetyltransferase-2 *(NAT-2)* and Cytochrome P-450 *(CYP2C9)* are other XMEs that are studied for genetic susceptibility for DNA damage due to pesticide exposure. Singh and coworkers studied polymorphism of these enzymes in workers exposed to organophosphate pesticides and revealed that DNA damage was higher in persons with one particular allele as compared to the persons with another allele [147]. Pregnant women exposed to heavy metals have been reported to have placental accumulation of these metals which affects the transport of nutrients from mother to fetus. Metallothionein is involved in micronutrient transport and detoxification of placental toxins. Polymorphism in this gene results in differential accumulation of cadmium in placenta [148]. Similarly, SNPs in metallothionein *(MT)* gene have also been shown to be responsible for varying susceptility to ALS. Antioxidant enzymes help in preventing the oxidative stress and SNPs related to these enzymes also showed varied response to environment. Superoxide dismutase *(SOD)* genotype reconstruction showed that *SOD1* (GG) and *SOD2* (GT) alleles decrease the risk of retinopathy of prematurity in preterm babies [149]. The above studies have elucidated that certain alleles involved in xenobiotics metabolism make individual more susceptible to diseases, who can be counseled to adopt preventive measures to protect themselves from adverse environmental influences.

## Conclusion

The present review emphasizes the importance of environmental cues and epigenetics on pathogenesis of neurodegenerative diseases. The role of early life exposure to environmental stimuli while ageing has largely remained underinvestigated which has been highlighted in this review. Present work postulates that the sporadic diseases can be considered as after effects of exposure in early life, in addition to prevalent theories of pathogenesis being investigated worldwide. Early life practices and environment determines physical and mental wellness in later stages due to genetic imprinting explained by epigenetics. Even though the bulk of research investigations have focused on molecular targets, the therapeutic outcome has not been very encouraging. A new focus on targeting the early life epigenetic mechanisms is imperative through larger studies. Whether developmental disorders and degenerative diseases have any epigenetic association could be revisited though launch of longitudinal animal studies. Therefore, prevention of disease by preempting early life exposure should be tested by launching worldwide public health initiatives. The mechanistic understanding of neurodegeneration provided in the review will likely provide new insights important for healthy lifestyle in the individuals at risk for such diseases.

## Competing interests

The authors declare that the research was conducted in the absence of any commercial or financial relationships that could be construed as a potential conflict of interest.

## Authors’ contribution

SM compiled the review of literature and wrote the manuscript, DKL provided the importance of pursuing early life exposure studies in degenerative diseases and called for review writing and edited the manuscript, VLS guided the first author in writing and compiling the manuscript; AA conceptualized the review writing, edited the manuscript and coordinated with various authors. All authors read and approved the final manuscript.

## Authors’ information

Shweta Modgil and Akshay Anand Equal first author.
